# Engagement Patterns and Clinical Outcomes Among Adolescents and Young Adults Enrolled in the Somethings Digital Peer Support Platform: Retrospective Observational Study

**DOI:** 10.2196/108377

**Published:** 2026-11-03

**Authors:** Kelsey McAlister, Landry Huffman, Courtney Jewell, Amber W Childs, Evelyn Cox, Jennifer Huberty

**Affiliations:** 1 Fit Minded, Inc, 2901 E Greenway Road, PO Box 30271, Phoenix, AZ, United States, 1 (602) 935-6986; 2 Somethings, New York City, NY, United States

**Keywords:** peer support, digital mental health, adolescent mental health, text-based support, rural mental health, measurement-based care

## Abstract

**Background:**

Adolescent mental health needs remain largely unmet, particularly among youth in geographically underserved communities. Digital peer support is a promising approach to expanding access to mental health support, yet real-world evidence on engagement patterns and clinical outcomes remains limited.

**Objective:**

The purpose of this study was to characterize engagement patterns and real-world outcomes among adolescents and young adults enrolled in a commercially available, text-based peer support platform.

**Methods:**

This retrospective observational study analyzed secondary data from 5673 adolescents and young adults enrolled in a commercially available digital peer support platform from January 29, 2023, through June 3, 2026. Engagement was operationalized as communication days per month. Standardized clinical outcome measures (Patient Health Questionnaire-9 [PHQ-9], Generalized Anxiety Disorder-7 [GAD-7], and World Health Organization-Five Well-Being Index [WHO-5]) were administered as part of routine measurement-based care. Ordinary least squares regression was used to examine predictors of engagement, and complete-case pre-post change scores were evaluated across 4 assessment windows among participants with paired assessments.

**Results:**

The sample was predominantly female (4600/5673, 81.1%) with a mean age of 16.4 (SD 2.1) years and moderate-to-severe symptom burden at baseline (PHQ-9: mean 12.6, SD 6.1; GAD-7: mean 10.7, SD 5.0; WHO-5: mean 44.8, SD 21.0). Of enrolled adolescents and young adults, 98.1% (5565/5673) had at least one recorded engagement with the platform, with a mean of 4.56 (SD 6.12) communication days per month. Female sex was a significant positive predictor of engagement intensity (β=.64, SE 0.22; *P*=.004). Hispanic and Latino adolescents and young adults engaged significantly more frequently than White or non-Hispanic adolescents and young adults (β=.61, SE 0.26; *P*=.02). Health Professional Shortage Area (HPSA) shortage score was significantly and positively associated with engagement intensity (β=.08, SE 0.02; *P*<.001). Baseline symptom severity was not associated with engagement intensity (β=.094, SE 0.23; *P*=.68). Among participants with complete baseline and follow-up data (1437/5673, 25.3%), an exploratory complete-case analysis found within-measure paired scores improved significantly from baseline to follow-up across the PHQ-9, GAD-7, and WHO-5. At last assessment, 31.9% (237/742), 36.6% (222/606), and 37.8% (168/442) of adolescents and young adults met clinically meaningful improvement thresholds on the PHQ-9, GAD-7, and WHO-5, respectively.

**Conclusions:**

Text-based peer support shows preliminary promise as a scalable approach to expanding mental health access for underserved adolescents, though clinical improvement estimates are based on the subpopulation exposed to the digital peer support platform and may not generalize to the full enrolled population. Prospective, controlled studies with more complete outcome ascertainment are needed to establish causal efficacy.

## Introduction

Adolescent mental health in the United States is a growing public health concern. Rates of depression and anxiety among adolescents and young adults have increased substantially in the past decade, with approximately 1 in 5 adolescents meeting criteria for a diagnosable mental health condition [1,2]. Despite this known concern, access to mental health services remains limited. Fewer than 50% of adolescents and young adults with mental health needs receive any form of treatment, and limited accessibility is further compounded by geographic barriers, workforce shortages, and stigma surrounding help-seeking behaviors [3,4]. Moreover, individuals in rural and underserved communities present with additional access barriers, with primary care and specialty mental health services often being unavailable within a reasonable geographic radius [5,6].

Digital mental health platforms are a promising approach for expanding treatment and services for adolescents and young adults who would otherwise remain underserved [7,8]. Among the available resources, peer-mentor support, in which individuals with shared lived experience provide structured, nonclinical support to adolescents and young adults, has gained traction as a developmentally appropriate and scalable approach for youth [9,10]. Peer-mentor relationships may be impactful due to their nonhierarchical design, which may reduce the perceived power differential that adolescents report as a barrier to engaging with formal clinical services [11]. Peer-mentor models have been associated with reductions in mental health symptom severity, improvements in help-seeking behaviors, and decreased feelings of isolation among youth [12-15]. Despite this promise, the evidence base for digitally delivered peer-mentor support among youth remains limited.

The platform under study is a commercially available digital mental health platform that delivers one-on-one peer mentorship to adolescents and young adults (aged 13‐26) via asynchronous text-based messaging. Text-based delivery aligns with the communication preferences of digitally native adolescents and young adults and has demonstrated feasibility as a modality for mental health service engagement in this population [16,17]. What remains unclear is how adolescents and young adults engage with text-based peer support in real-world environments and whether engagement is associated with measurable symptom change.

The purpose of this study was to characterize engagement patterns and real-world outcomes among adolescents and young adults enrolled in a commercially available, text-based peer support platform. Using retrospective data from a large naturalistic sample, we aimed to achieve three objectives: (1) to describe the demographic and clinical characteristics of enrolled adolescents and young adults, and the patterns of platform engagement; (2) to identify demographic and clinical predictors of engagement intensity; and (3) to describe within-measure pre-post changes in depressive symptoms, anxiety symptoms, and well-being among adolescents and young adults with paired assessments. Findings from this study contribute real-world evidence on how adolescents and young adults engage with digital peer support and provide insight into its potential to support improvements in mental health and well-being.

## Methods

### Study Design and Participants

This retrospective study analyzed secondary data from Somethings, a commercially available digital mental health platform delivering one-on-one peer mentorship to adolescents and young adults. The dataset included self-reported clinical outcome data and platform engagement metrics, collected as part of routine measurement-based care activities within the Somethings platform. Adolescents and young adults were included if they were enrolled in the Somethings platform during the study period and had demographic data recorded. Every enrolled adolescent and young adult was matched with a peer mentor. Eligible participants were identified through a retrospective cohort sampling approach from backend platform records, spanning from January 29, 2023, through June 3, 2026 (ie, the data were extracted). No direct recruitment or prospective enrollment occurred. This study was conducted and reported in accordance with the STROBE (Strengthening the Reporting of Observational Studies in Epidemiology) guidelines for observational cohort studies (Checklist 1).

### Ethical Considerations

The study protocol was reviewed by BRANY (Biomedical Research Alliance of New York) Institutional Review Board (study ID: 26-138-1708) and was determined to qualify for exemption under 45 CFR 46.104(d)(4) [18] which governs secondary analysis of existing data. Because the study used retrospective, deidentified data and involved no direct participant contact, informed consent procedures were not required. All data were deidentified prior to transfer to the research team, and no individually identifiable information was accessible during analysis. Participants did not receive compensation for inclusion in this research.

### Text-Based Peer Support Platform (Somethings)

Somethings is a commercially available digital mental health platform accessible via iOS and Android mobile apps. Somethings is delivered through partnerships with payers and public-sector entities at no cost to the end user. Adolescents and young adults (aged 13‐26) create an account, complete a structured intake assessment, and are shown 3 to 5 certified peer specialist mentor options matched to their reported interests, demographics, and presenting concerns, from which they select the mentor with whom they want to work.

Mentors at Somethings are young adults with their own lived mental health experience who complete structured training to become certified peer specialists, which includes coursework in lived-experience–informed support, crisis recognition, ethical boundaries, and communication skills, followed by a certification exam administered by the relevant state or credentialing body. All mentors operate under structured clinical oversight, with licensed clinical staff available for case consultation and escalation of safety concerns. The primary modality of service delivery is asynchronous text-based messaging, with video calls available as a supplementary engagement channel. Consistent with measurement-based care principles [19], Somethings administers standardized clinical outcome assessments at enrollment and at defined follow-up intervals throughout each adolescent and young adult’s engagement with the platform.

### Study Measures

At enrollment, adolescents and young adults completed a structured intake assessment capturing age, sex, race and ethnicity, and zip code. As part of the deidentification process, zip codes were not shared with the study team. Instead, participants were assigned Health Professional Shortage Area (HPSA) scores based on their zip codes prior to data transfer. HPSA scores are a federal designation used to quantify the degree of mental health provider shortage in a given geographic area [20], with higher scores indicating greater shortage and underservice.

Standardized clinical outcome assessments were administered as part of routine measurement-based care. The intake survey was delivered on the second day of app use, reflecting a deliberate design decision to reduce onboarding friction while maintaining proximity to enrollment. Follow-up surveys were administered at 30-day intervals and were sent only to adolescents and young adults who had messaged on at least one distinct day in the preceding 30-day period. Because of this conditional routing, all subsequent clinical outcome findings reflect only participants who remained actively engaged and should not be interpreted as generalizable to the full enrolled population. Adolescents and young adults were routed to 1 of 3 outcome measures (ie, Patient Health Questionnaire-9 [PHQ-9], Generalized Anxiety Disorder-7 [GAD-7], and World Health Organization-Five Well-Being Index [WHO-5]) based on their response to a single screening item asking which emotional experience most closely matched their current state (feeling down or low energy, stressed or anxious, or feeling okay overall; Supplemental Material 1 in Multimedia Appendix 1). During a period in 2024, adolescents and young adults were able to select multiple responses to this screening item and could therefore be routed to more than one outcome measure, which may account for discrepancies in sample sizes.

### Depression

Depressive symptom severity was assessed using the PHQ-9 [21], a 9-item self-report measure scored 0 to 27, with higher scores indicating greater symptom severity. The PHQ-9 demonstrates strong internal consistency (Cronbach α=0.89) and good test-retest reliability (*r*=0.84) [21]. Standard clinical thresholds were applied: minimal (0‐4), mild (5-9), moderate (10-14), moderately severe (15-19), and severe (20-27). Clinically meaningful improvement was defined as a reduction of 5 or more points from baseline [21,22].

### Anxiety

Anxiety symptom severity was assessed using the GAD-7 [23], a 7-item self-report measure scored 0 to 21, with higher scores indicating greater severity. The GAD-7 demonstrates strong internal consistency (Cronbach α=0.92) and good test-retest reliability (intraclass correlation=0.83) [23]. Standard thresholds were applied: minimal (0‐4), mild (5-9), moderate (10-14), and severe (15-21). Clinically meaningful improvement was defined as a reduction of 4 or more points from baseline [22,23].

### Well-Being

Subjective well-being was assessed using the WHO-5 [24], a 5-item self-report measure scored 0 to 100, with higher scores indicating better well-being. A raw score below 50 is indicative of poor well-being [25]. Clinically meaningful improvement was defined as an increase of 10 or more points from baseline [24,26].

### Engagement Variables

The primary engagement metric was communication days per month, calculated as the total number of days an adolescent/young adult communicated with their mentor, divided by the total number of months of observed engagement. Participants whose enrollment coincided with the data extraction date were excluded from engagement-rate calculations, as their single reporting period was 0 days, precluding a valid monthly rate. These participants remained in all other analyses.

### Statistical Analyses

Descriptive statistics were used to characterize the sample. Continuous variables are summarized using means and SDs, and categorical variables are summarized using frequencies and percentages. Chi-square tests and independent-samples 2-tailed *t* tests were used, as appropriate, to evaluate whether the demographic composition differed between enrolled and engaged subsamples. To evaluate informative dropout, baseline severity scores were compared between adolescents and young adults who completed at least one follow-up assessment and those who did not, using independent-samples 2-tailed *t* tests and logistic regression with dropout as the outcome variable.

To examine demographic and clinical predictors of platform engagement, ordinary least squares regression was used. Two models were estimated: model A included age, sex, race/ethnicity, and HPSA score and was estimated in the subset of adolescents and young adults with available location data; Model B added baseline symptom severity to the demographic predictors. Baseline clinical severity was operationalized as a composite *z* score averaging standardized PHQ-9, GAD-7, and reverse-scored WHO-5 scores, with higher values indicating greater symptom burden. To preserve the validity of the *z* score as a genuine multi-instrument composite, model B was restricted to adolescents with at least 2 of these 3 baseline measures nonmissing, as adolescents with only a single baseline measure would have a z score value reflecting 1 instrument alone rather than a true composite. Sensitivity analyses were conducted to restrict model B to adolescents with all 3 measures and to those with at least one measure. Sensitivity analyses were also conducted for both models, including heteroskedasticity-robust SEs (HC3) and log-transformation of the outcome variable (log1p), to assess the robustness of findings to violations of homoskedasticity and nonnormality. Models were estimated using dummy-coded indicators for race/ethnicity, with White non-Hispanic adolescents and young adults specified as the reference category based on subgroup size (the largest group in the sample), consistent with standard practice for maximizing precision of estimated contrasts. Because reference group encoding using the majority group as the implicit comparator has been critiqued as a source of interpretive bias in race/ethnicity research [27], a sensitivity check was reestimated using an alternate reference category (Asian adolescents and young adults), selected by alphabetical ordering of racial/ethnic categories, a convention sometimes used to avoid discretionary selection of the reference group.

To describe within-measure symptom change among adolescents and young adults with paired assessments, pre-post change scores were computed as the difference between each participant’s follow-up and baseline score on each measure. Change from baseline was evaluated across 4 time windows: 30 days, 8 weeks, 12 weeks, and the last available assessment. One-sample 2-tailed *t* tests were used to evaluate whether the mean change differed significantly from 0. To illustrate the full picture of symptom change and improvement, several types of change were included in the analyses: proportion of adolescents and young adults achieving clinically meaningful improvement, proportion moving down a symptom severity category, proportion showing any improvement (ie, any decrease in PHQ-9 or GAD-7 scores, or any increase in WHO-5), and proportion showing a minimum clinically important difference (MCID) between any 2 chronologically ordered assessments. Clinically meaningful improvement was calculated using established MCID thresholds: a reduction of 5 points or more for PHQ-9, a reduction of 4 points or more for GAD-7, and an increase of 10 points or more for WHO-5.

A formal power analysis was not conducted, as the retrospective nature of this study precluded prospective sample size determination. Instead, the analytic samples reflected the full population of eligible adolescents and young adults available within the observation window for each aim, and they varied based on data availability. All analyses were conducted using R (version 4.3.2; R Foundation for Statistical Computing).

## Results

### Data Availability

The dataset comprised 5673 adolescents and young adults (aged 13‐26) enrolled in the platform from January 29, 2023, through June 3, 2026. Of these, 2943 completed a baseline survey, which was not administered throughout the full observation window; they comprise the completed-baseline sample. A total of 1437 (25.3% of the enrolled cohort) adolescents and young adults had both a labeled baseline assessment and at least one follow-up assessment on any outcome measure, constituting the survey-paired analytic sample used for the exploratory complete-case clinical outcome analyses (Figure 1). Paired sample sizes varied by outcome measure and assessment window, reflecting both conditional survey routing and availability of valid baseline and follow-up scores. The maximum instrument-specific paired samples were PHQ-9 (n=742), GAD-7 (n=606), and WHO-5 (n=442).

**Figure 1.**
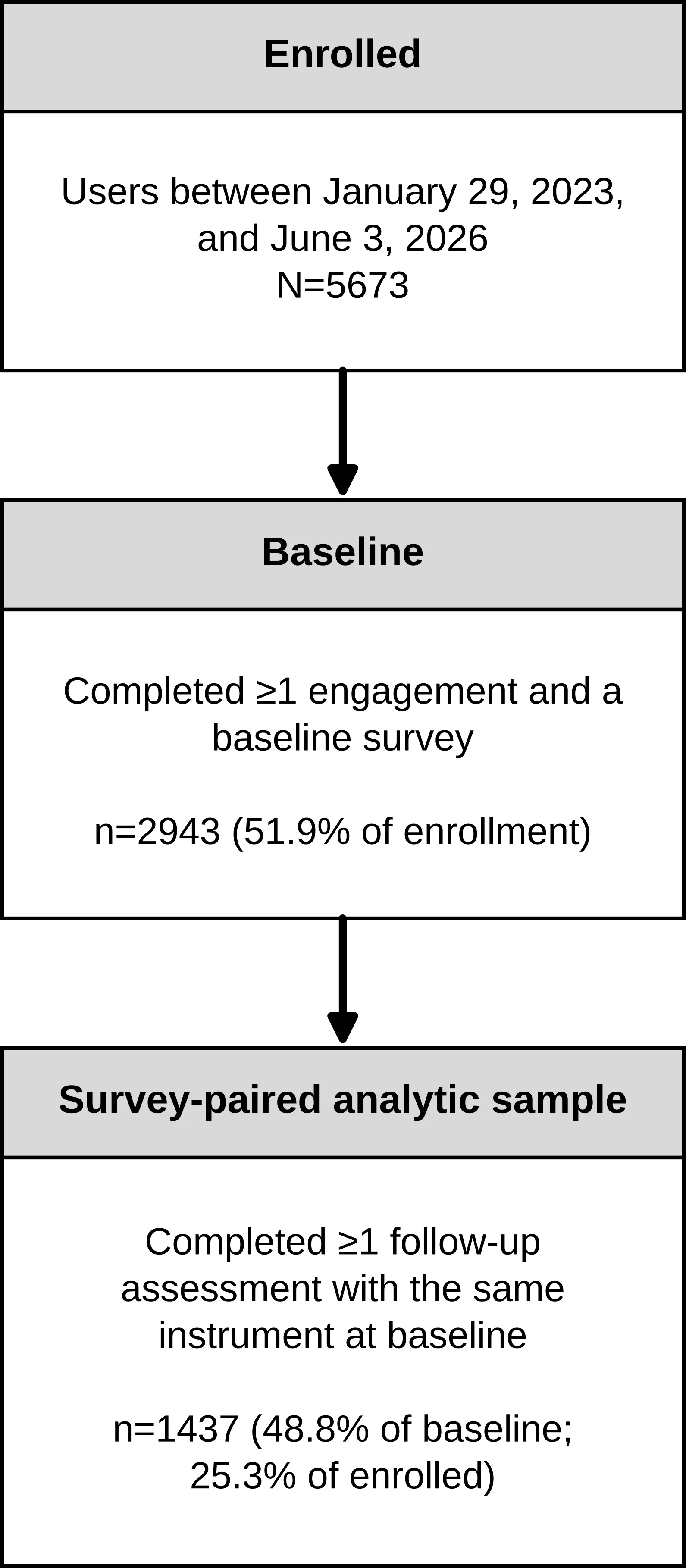
User-flow diagram of adolescents and young adults enrolled in a text-based peer-support platform, from enrollment through follow-up survey completion. The survey-paired analytic sample included adolescents and young adults with a baseline assessment and at least one follow-up assessment on the same outcome measure. Instrument-specific paired samples were Patient Health Questionnaire-9 (PHQ-9; n=742), Generalized Anxiety Disorder-7 (GAD-7; n=606), and World Health Organization-Five Well-Being Index (WHO-5; n=442).

### Aim 1: Demographic and Clinical Characteristics and Engagement Patterns

Demographic characteristics across the enrolled population, those who completed a baseline assessment, and the survey-paired analytic sample are presented in Table 1. Among the 5673 enrolled participants, the population was predominantly female (n=4600, 81.1%), with a mean age of 16.4 (SD 2.1; median 16.0, IQR 15-18; range 13‐26) years. By race and ethnicity, 42.9% (n=2435) identified as White or non-Hispanic, 22.9% (n=1298) as Black or African American, 13.0% (n=735) as Hispanic or Latino, 4.3% (n=243) as Asian, and 10.7% (n=607) as other or unknown identity. Geographic underservice data, indexed by HPSA shortage score, were available for 5535 (97.6%) enrolled adolescents and young adults; among those with data, the mean shortage score was 1.9 (SD 5.0). At baseline, adolescents and young adults presented with moderate depressive (mean 12.6, SD 6.1; n=1659) and anxiety (mean 10.7, SD 5.0; n=1571) symptoms and poor well-being (mean 44.8, SD 21.0; n=1275), on average. Female adolescents and young adults presented with significantly higher depressive and anxiety symptom severity and lower well-being than male adolescents and young adults: mean PHQ-9 of 12.8 (SD 6.0) vs 11.3 (SD 6.2; *P*<.001), mean GAD-7 of 10.9 (SD 5.0) vs 9.9 (SD 5.1; *P*=.01), and mean WHO-5 of 43.3 (SD 20.57) vs 51.7 (SD 21.9; *P*<.001).

Of the 5673 enrolled adolescents and young adults, 5565 (98.1%) had at least one recorded engagement with the platform. The mean number of communication days per month was 4.56 (SD 6.12; median 1.93, IQR 0.54‐6.34), reflecting a right-skewed distribution. Engagement descriptives by sex and race/ethnicity are presented in Table 2.

The survey-paired analytic sample differed modestly from the enrolled sample in racial composition (*χ*²_4_=13.11; *P*=.01), with Black/African American, Hispanic/Latino, other/unknown, and White participants showing approximately 10% more attrition than Asian participants. HPSA shortage scores and the distributions of sex did not significantly differ across enrolled and survey-paired samples.

**Table 1.** Demographic and baseline clinical characteristics of enrolled adolescents and young adults.

Variable	All enrolled (n=5673)	Completed baseline (n=2943^a^)	Survey-paired (n=1437^a^)
Age (years)^b^, mean (SD); median (IQR); range	16.4 (2.1); 16.0 (15-18); 13‐26	16.3 (1.9); 16.0 (15-17); 13‐26	16.4 (1.9); 16.0 (15-18); 13‐25
Sex, n (%)			
Female	4600 (81.1)	2483 (84.4)	1177 (81.9)
Male	908 (16.0)	449 (15.3)	222 (15.4)
Missing	165 (2.9)	11 (0.4)	38 (2.6)
Race/ethnicity^c^, n (%)			
Asian	243 (4.3)	158 (5.4)	79 (5.5)
Black/African American	1298 (22.9)	667 (22.7)	335 (23.3)
Hispanic/Latino	735 (12.9)	367 (12.5)	168 (11.7)
Other/unknown	607 (10.7)	316 (10.7)	136 (9.5)
White (non-Hispanic)	2435 (42.9)	1352 (45.9)	643 (44.7)
Missing	355 (6.3)	83 (2.8)	76 (5.3)
HPSA^d^ shortage score, mean (SD); n with data	1.9 (5.0); 5535	1.5 (4.5); 2928	1.4 (4.4); 1431
Baseline clinical scores^e^, mean (SD); n with data			
PHQ-9^f^ (0‐27)	12.6 (6.1); 1659	12.6 (6.1); 1659	12.4 (6.1); 838
GAD-7^g^ (0‐21)	10.7 (5.0); 1571	10.7 (5.0); 1571	10.8 (4.9); 780
WHO-5^h^ (0‐100)	44.8 (21.0); 1275	44.8 (21.0); 1275	44.5 (20.8); 588

a Completed baseline and survey-paired groups are overlapping analytic samples, not mutually exclusive groups, so they do not sum to 5673. Survey-paired participants are also in the completed baseline group. The exception is 60 participants with a baseline survey without a valid PHQ, GAD, or WHO score; these participants don't contribute to any within-measure change analysis.

b Age restricted to valid range (13‐26 y); values outside this range treated as missing.

c Race/ethnicity was assessed by one, single-response question.

d HPSA: Health Professional Shortage Area.

e “n” reflects adolescents and young adults with a score on that measure at their first labeled baseline survey. Identical values in the “all enrolled” and “completed baseline” columns reflect that baseline scores are available only for those who completed a baseline survey.

f PHQ-9: Patient Health Questionnaire-9.

g GAD-7: Generalized Anxiety Disorder-7.

h WHO-5: World Health Organization-Five Well-Being Index.

**Table 2.** Mean communication days per month with peer mentors, by demographic group, among enrolled adolescents and young adults^a^.

Variable	n	Mean (SD)	Median (IQR)
Overall^b^	5565	4.56 (6.12)	1.93 (0.54‐6.34)
Sex			
Male	906	3.98 (6.15)	1.39 (0.44‐5.25)
Female	4591	4.67 (6.13)	2.01 (0.55‐6.59)
Missing	68	4.69 (5.03)	2.89 (1.15-6.35)
Race/ethnicity			
Asian	242	4.76 (5.60)	2.40 (0.53‐6.88)
Black/African American	1295	4.69 (6.30)	1.81 (0.55‐6.11)
Hispanic/Latino	735	5.08 (6.76)	2.33 (0.66‐7.56)
Other/unknown	607	4.34 (6.42)	1.71 (0.50‐5.82)
White (non-Hispanic)	2428	4.39 (5.84)	1.91 (0.52‐6.24)
Missing	258	4.30 (5.51)	2.14 (0.61-5.49)

a Values are communication days per month. Distribution is right-skewed; median and IQR are the primary descriptive statistics.

b “Overall” row reflects all enrolled adolescents and young adults with at least one engagement record (5565/5673; 98.1% of enrolled).

### Aim 2: Predictors of Engagement Intensity

Ordinary least squares regression was used to examine demographic and clinical predictors of communication days per month. In model A (n=5453), female adolescents and young adults communicated 0.64 more days per month than male adolescents and young adults (β=.64, SE 0.22; *P*=.004). Hispanic/Latino adolescents and young adults communicated significantly more frequently than White or non-Hispanic adolescents and young adults (β=.61, SE 0.26; *P*=.02). No other racial/ethnic group differed significantly from White or non-Hispanic adolescents. Communication days did not differ significantly across ethnic groups when using Asian race as the reference category (Supplemental Material 2 in Multimedia Appendix 1). Age was not significantly associated with communication days (β =−.05, SE 0.04; *P*=.23). HPSA shortage score was significantly and positively associated with communication days (β=.08, SE 0.02; *P*<.001), such that adolescents and young adults residing in areas with greater mental health care shortages communicated more frequently with their mentors. Model A explained little variance in engagement (*R*²=.008; Table 3). The sex (female), race/ethnicity (Hispanic/Latino), and HPSA effects were consistent under a heteroskedasticity-robust specification and with a log-transformation of the outcome (Supplemental Material 3 in Multimedia Appendix 1).

**Table 3.** Ordinary least squares regression results for demographic and clinical predictors of monthly communication days among enrolled adolescents and young adults.^a^

Variable	Model A (n=5453)^b^		Model B (n=1080)^c^	
	β (SE)	*P* value	β (SE)	*P* value
Age (years, centered)	−.05 (0.04)	.23	.03 (0.10)	.76
Sex (reference: male)				
Female	.64 (0.22)	.004	.11 (0.50)	.83
Race/ethnicity (reference: White/non-Hispanic)				
Asian	.49 (0.42)	.24	2.02 (0.88)	.02
Black/African American	.27 (0.21)	.21	.82 (0.46)	.07
Hispanic/Latino	.61 (0.26)	.02	−.27 (0.63)	.67
Other/unknown	−.11 (0.25)	.65	.01 (0.61)	.99
HPSA^d^ shortage score	.08 (0.02)	<.001	.04 (0.05)	.43
Baseline severity (*z* score)	—^e^	—	.09 (0.23)	.68

a Model A restricted to adolescents and young adults with available HPSA shortage score data. Model B was restricted to adolescents and young adults with at least 2 of 3 baseline clinical scores and engagement data; baseline severity is a composite *z* score (Patient Health Questionnaire-9 [PHQ-9], Generalized Anxiety Disorder-7 [GAD-7], and World Health Organization-Five Well-Being Index [WHO-5] reversed), with higher values indicating greater symptom burden.

b *R*2=0.008.

c *R*2=0.009.

d HPSA: Health Professional Shortage Area.

e Not applicable.

Model B examined baseline clinical severity alongside demographics and HPSA score (n=1080). Baseline symptom severity was not a significant predictor of communication days per month (β=.09, SE 0.23; *P*=.68). HPSA shortage score was also not a significant predictor once baseline severity was included in the model (β=.04, SE 0.05; *P*=.43). Asian adolescents and young adults communicated more frequently than White non-Hispanic adolescents and young adults (β=2.02, SE 0.88; *P*=.02); Black/African American adolescents and young adults showed a similar but nonsignificant trend (β=.83, SE 0.46; *P*=.07). No other demographic predictors reached significance. The null severity and HPSA effects were consistent across the heteroskedasticity-robust and log-transformed specifications (Supplemental Material 3 in Multimedia Appendix 1). Sensitivity analyses examining alternative thresholds for baseline severity measurement completeness are reported in Supplemental Material 4 in Multimedia Appendix 1.

### Aim 3: Within-Measure Pre-Post Change Scores

Findings in this section reflect only the subset of participants who remained engaged enough to complete follow-up assessments. Pre-post change scores were examined at 4 assessment windows: first follow-up (median of 36 d, IQR 26-62 postbaseline for the PHQ-9; 30 d, IQR 16-41 for the GAD-7; and 20 d, IQR 15-36 for the WHO-5), 8-week, 12-week, and last assessment (median of 103 d, IQR 42-174 for the PHQ-9; 66.5 d, IQR 33-192 for the GAD-7; and 65 d, IQR 33-178 for the WHO-5). Baseline symptom severity did not differ significantly between adolescents and young adults who completed at least one follow-up assessment and those who did not on any of the 3 measures (PHQ-9: *P*=.15; GAD-7: *P*=.68; WHO-5: *P*=.69). Follow-up surveys were administered only to adolescents and young adults who had communicated with their mentor during the preceding 30-day period.

Across all 3 measures, statistically significant improvement from baseline was observed at all 4 assessment windows (all *P*<.001; Table 4). Within-measure paired scores improved by the first follow-up and were sustained across all subsequent windows. Peak improvement was observed at the 8-week window for GAD-7 and PHQ-9, and at the 12-week window for WHO-5. The mean PHQ-9 decreased by 2.0 points at the first follow-up (n=742), with a similar decline of 2.0 points at the 8-week follow-up (n=284) and 1.9 points at the 12-week follow-up. The mean GAD-7 declined by 2.1 points at the first follow-up (n=606), 2.4 points at the 8-week follow-up (n=212), and 2.3 points at the 12-week follow-up. The mean WHO-5 improved by 6.3 points at the first follow-up (n=442), 8.5 points at the 8-week follow-up, and 10.5 points at the 12-week follow-up (n=148).

**Table 4.** Within-measure pre-post change in depression (Patient Health Questionnaire-9 [PHQ-9]), anxiety (Generalized Anxiety Disorder-7 [GAD-7]), and well-being (World Health Organization-Five Well-Being Index [WHO-5]) scores by assessment window among enrolled adolescents and young adults with paired survey data.^a^

Window	Participants, n	Baseline, mean (SD)	Follow-up, mean (SD)	Change, mean (SD; 95% CI)	MCID^b^, n/N (%)^c^
PHQ-9 (0‐27; MCID ≥5-point decrease)					
First follow-up (median 36 d, IQR 26-62)	742	12.6 (6.1)	10.6 (6.4)	−2.0 (5.7; −2.40 to −1.58)	223/742 (30.1)
8-week	284	12.7 (6.0)	10.7 (6.5)	−2.0 (5.7; −2.70 to −1.38)	84/284 (29.6)
12-week	348	12.4 (6.4)	10.5 (6.8)	−1.9 (6.2; −2.54 to −1.24)	103/348 (29.6)
Last assessment (median 103 d, IQR 42-174)	742	12.6 (6.1)	10.4 (6.6)	−2.1 (6.0; −2.57 to −1.70)	237/742 (31.9)
GAD-7 (0‐21; MCID ≥4-point decrease)					
First follow-up (median 30 d, IQR 16-41)	606	10.9 (4.8)	8.8 (5.3)	−2.1 (5.0; −2.51 to −1.71)	225/606 (37.1)
8-week	212	11.4 (4.9)	9.0 (5.5)	−2.4 (5.3; −3.13 to −1.69)	81/212 (38.2)
12-week	192	11.2 (4.9)	9.0 (5.3)	−2.3 (5.2; −3.01 to −1.51)	70/192 (36.5)
Last assessment (median 66.5 d, IQR 33-192)	606	10.9 (4.8)	8.9 (5.5)	−2.0 (5.4; −2.47 to −1.61)	222/606 (36.6)
WHO-5 (0‐100; MCID ≥10-point increase)					
First follow-up (median 20 d, IQR 15-36)	442	45.4 (21.1)	51.6 (23.3)	6.3 (19.1; 4.49 to 8.06)	169/442 (38.2)
8-week	154	43.1 (20.0)	51.6 (23.8)	8.5 (22.6; 4.93 to 12.11)	66/154 (42.9)
12-week	148	42.8 (20.3)	53.2 (24.5)	10.5 (21.9; 6.91 to 14.01)	64/148 (43.2)
Last assessment (median 65 d, IQR 33-178)	442	45.4 (21.1)	51.1 (24.7)	5.8 (20.9; 3.88 to 7.78)	168/442 (38.0)

a All one-sample 2-tailed *t* tests vs zero: *P*<.001 at every window. Median days shown for “first follow-up” and “last assessment”; timing varies by measure, reflecting differences in which instrument each adolescent was routed to at a given survey cycle.

b MCID: minimum clinically important difference.

c Proportion of adolescents and young adults with paired data meeting or exceeding the MCID for each instrument at that window.

Table 5 shows the proportions of participants with paired data who showed improvement by type of improvement. Between the first and last assessments, clinically meaningful improvement was observed in at least one-third of adolescents and young adults with paired data: 31.9% (237/742) met the PHQ-9 MCID of a decrease of 5 points or more, 36.6% (222/606) met the GAD-7 MCID of a decrease of 4 points or more, and 38.0% (168/442) met the WHO-5 MCID of an increase of 10 points or more. Nearly half of participants with paired data moved down a symptom severity category on the PHQ-9 (336/742, 45.3%) and GAD-7 (274/606, 45.2%), and between 48.9% (413/844) and 60.2% (289/480) experienced an MCID improvement between any 2 consecutive assessments for at least one symptom. Of the paired sample, 816 out of 1207 (67.6%) improved on at least one measure (ie, PHQ-9, GAD-7, and WHO-5) from preassessment to postassessment. As a sensitivity analysis, MCID rates were recalculated against the full enrolled cohort (N=5673) under conservative and liberal bounding assumptions for participants without paired data. Assuming no improvement, estimated rates ranged from 3.0% (168/5673) to 4.2% (237/5673); assuming improvement, they ranged from 77.6% (4405/5673) to 78.8% (4473/5673). These compared with 31.9% (237/742) to 38.0% (168/442) in the complete-case analysis (Supplemental Material 5 in Multimedia Appendix 1).

**Table 5.** Proportion of adolescents and young adults with paired survey data meeting clinically meaningful improvement thresholds on the Patient Health Questionnaire-9 (PHQ-9), Generalized Anxiety Disorder-7 (GAD-7), and World Health Organization-Five Well-Being Index (WHO-5) by assessment window.^a^

Symptom	MCID^b^ improvement^c^, n/N (%)	Moved down a severity category^c^, n/N (%)	Any positive improvement^c^, n/N (%)	MCID improvement, any assessments^d^, n/N (%)
PHQ-9	237/742 (31.9)	336/742 (45.3)	453/742 (61.1)	413/844 (48.9)
GAD-7	222/606 (36.6)	274/606 (45.2)	374/606 (61.7)	360/654 (55.0)
WHO-5	168/442 (38.0)	122/422 (28.9)	260/442 (58.8)	289/480 (60.2)

a Overall, 67.6% (816/1207) of the paired sample showed improvement in at least one of the PHQ-9, GAD-7, or WHO-5 scores from preassessment to postassessment. Additionally, 42.0% (507/1207) of the paired sample achieved an MCID in at least one of these measures between the first and last available assessments.

b MICD: minimum clinically important difference.

c From the first to the last available assessment.

d MCID improvement occurring between any 2 chronologically ordered assessments.

## Discussion

### Overview

The purpose of this study was to characterize engagement patterns and real-world clinical outcomes among adolescents and young adults enrolled in a commercially available text-based peer support platform. Using retrospective data from a large naturalistic sample, we aimed to achieve three objectives: (1) to describe the demographic and clinical characteristics of enrolled adolescents and young adults and their patterns of platform engagement; (2) to identify demographic and clinical predictors of engagement intensity; and (3) to describe within-measure pre-post changes in depressive symptoms, anxiety symptoms, and well-being among adolescents and young adults with paired assessments. Three primary findings emerged. First, the platform demonstrated broad real-world reach, enrolling a racially and ethnically diverse sample of adolescents and young adults presenting with moderate-to-severe mental health symptom burden. Second, female adolescents and young adults and those residing in areas with greater mental health care shortages engaged most intensively with the platform, while baseline symptom severity was not associated with engagement intensity. Third, in an exploratory complete-case analysis of the retained subcohort with paired assessments, adolescents and young adults demonstrated statistically significant within-measure score improvements, with at least one-third of this subcohort achieving clinically meaningful improvement on each measure (ie, PHQ-9, GAD-7, and WHO-5), and around two-thirds experiencing any positive improvement. These findings reflect the retained, actively engaged subcohort and should be interpreted as preliminary associations, since this study also lacks a control group.

### Platform Engagement and Accessibility

The peer support platform enrolled a racially and ethnically diverse sample of adolescents and young adults presenting with a meaningful mental health burden, with mean PHQ-9 and GAD-7 scores falling in the moderate range at baseline and mean WHO-5 scores indicative of poor well-being. This clinical profile is consistent with, and in some respects more severe than, profiles reported in studies of other digital mental health platforms serving adolescents, where baseline PHQ-9 scores in the moderate-to-moderately–severe range have been commonly observed [28,29], suggesting that peer support platforms may have the ability to reach adolescents and young adults with genuine clinical need rather than a subclinical population. The racial and ethnic composition of the enrolled sample, approximately 22.9% (1298/5673) Black or African American and 12.9% (735/5673) Hispanic or Latino, reflects greater diversity than samples typically reported in digital mental health trials, where racially and ethnically minoritized youth remain substantially underrepresented [30]. This pattern may reflect the accessibility advantages of text-based, asynchronous delivery, which removes common structural barriers, including scheduling constraints, transportation burden, and the perceived power differential that adolescents and young adults frequently cite as a deterrent to formal clinical services [11]. Notably, female adolescents and young adults presented with significantly higher symptom severity than male adolescents and young adults across all 3 measures, consistent with well-established sex differences in internalizing symptom expression during adolescence [31]. The predominantly female enrolled sample likely reflects both this elevated burden and the well-documented tendency of male adolescents and young adults to underuse mental health services, with systematic review evidence identifying masculine norms, self-stigma, and barriers to disclosure as primary drivers of reduced help-seeking in this group [32]. This finding is congruent with ongoing challenges observed in digital health platforms among male users. Future research should examine the impact of platform design features on reach among historically underserved groups, including whether the existing demographic matching algorithm (eg, sex, race/ethnicity) meaningfully improves engagement or outcomes relative to nonconcordant matches.

### Sociodemographic Differences in Engagement Intensity

Adolescents and young adults residing in communities with greater mental health care provider shortages, indexed by HPSA score, communicated significantly more frequently with their mentors, and this finding held robustly across model specifications. This pattern is consistent with previous studies that have identified increased need for and use of digital health care services among those residing in geographically underserved communities [33,34]. Several mechanisms may explain this association, including greater unmet mental health need, reduced access to alternative sources of support, or increased reliance on digital resources in areas with limited behavioral health infrastructure. Because service use data were not available, it is not possible to determine whether digital peer support functioned as a substitute for, complement to, or an independent source of support relative to traditional mental health services. These findings may have direct relevance for policy discussions about the role of digital peer support in expanding behavioral health infrastructure in underserved communities, where provider-to-population ratios for mental health care fall well below federal adequacy thresholds [35]. Female adolescents and young adults communicated significantly more frequently than male adolescents and young adults, consistent with the well-established pattern of greater mental health service engagement among girls and young women [32]. Hispanic and Latino adolescents and young adults also communicated more frequently than White non-Hispanic adolescents and young adults, a finding that is noteworthy given national data indicating that Hispanic and Latino youth have substantially lower rates of digital mental health service use compared to White peers [36]. However, this contrast did not hold under an alternate reference category specification. Given this sensitivity to the choice of reference group, this finding should be considered preliminary despite its robustness to heteroskedasticity-robust and log-transformed specifications. However, when baseline symptom severity was added to the model, Asian adolescents and young adults emerged as communicating significantly more frequently than White non-Hispanic adolescents and young adults, a contrast that was not significant in the model without severity included. This finding emerged only in the smaller severity-restricted subsample, and additional testing is needed to confirm its robustness before it can be considered more than preliminary. Baseline symptom severity was not a significant predictor of communication days per month, indicating that adolescents and young adults across the full range of presenting symptom severity engaged at comparable rates and that elevated symptom burden alone does not preclude platform use. This null finding suggests that raw engagement frequency may not capture the full spectrum of how adolescents with different symptom burdens use the platform. Future research should examine engagement quality or trajectory instead of frequency alone and identify which mentor behaviors sustain engagement among the highest-need subgroups.

### Clinical Symptom Improvements

In this exploratory complete-case analysis, adolescents and young adults enrolled on the platform who contributed paired assessments demonstrated statistically significant within-measure score changes in depression, anxiety, and well-being that emerged by the first follow-up assessment and were sustained across all subsequent observation windows through a median of 103 (IQR 42-174) days postenrollment. These findings reflect the retained subcohort of adolescents and young adults with sufficient engagement to complete follow-up surveys and should not be generalized to the full enrolled population as evidence of platform-wide effectiveness, though the survey threshold was designed to ensure follow-up assessment occurred only among those with meaningful exposure to the platform. However, the observed improvements are broadly consistent with outcome trajectories reported in the peer support literature for adolescents and young adults, where reductions in depressive and anxiety symptom severity have been observed across a range of modalities and delivery formats [37,38]. However, this result warrants careful interpretation. Mean change scores across all 3 measures, while statistically significant, fell below established MCID thresholds, with mean PHQ-9 and GAD-7 reductions of approximately 2 points at each observation window falling short of the 5-point and 4-point MCID criteria, respectively. This gap between statistical and clinical significance is not uncommon in real-world observational studies of naturalistic samples, where heterogeneity in engagement, symptom severity, and duration of use attenuates mean effect sizes relative to efficacy trials with controlled conditions and protocol-driven adherence [29]. Additionally, these findings cannot be solely attributed to the platform in the absence of a control condition.

More informative than mean change scores in the present context are the proportions of adolescents and young adults achieving clinically meaningful improvement, with 31.9% (237/742) meeting the PHQ-9 MCID, 36.6% (222/606) meeting the GAD-7 MCID, and 38.0% (168/442) meeting the WHO-5 MCID at last assessment. These proportions are consistent with effect sizes reported in the broader peer support literature for youth, where improvements in depression and anxiety symptoms are typically modest in magnitude but meaningful in a clinical context, particularly given the nonclinical, nonprotocol-driven nature of peer-delivered support relative to structured therapeutic interventions [37,38]. In the absence of a control condition, it is not possible to determine the extent to which the observed improvements were attributable to the platform. Other factors, including natural symptom fluctuations, other supports, and regression to the mean may have contributed to the changes observed over time. Future studies using waitlist control or matched comparison designs are needed to isolate the contribution of the platform from other supports. These clinical outcomes present preliminary evidence of a directionally favorable signal in this retained, actively engaged subcohort, one that is sufficient to motivate more rigorous prospective evaluation but not to support causal claims about platform-wide efficacy.

### Implications for Digital Peer Support

These findings should be considered within the broader context of digital peer support as a category of intervention. The engagement and outcome patterns observed here are broadly consistent with those reported across other peer-delivered, digitally mediated support models [37,38], suggesting that the mechanisms this approach relies on may generalize beyond any single platform. Positioning this kind of peer support within a stepped-care model, as an accessible entry point rather than a substitute for clinical treatment when clinical need is indicated, may be a more appropriate framing for the field than evaluating individual implementations in isolation. However, more research is needed to determine which specific design elements, such as asynchronous delivery, near-peer matching, and message-based formats, actually drive engagement and outcomes across different peer support implementations, so that findings can be compared across models rather than evaluated one platform at a time.

### Strengths and Limitations

This study has several strengths. The sample size of 5673 enrolled adolescents and young adults represents one of the larger naturalistic cohorts reported in the peer support literature for this age group, enhancing the statistical precision of engagement estimates and the generalizability of demographic findings. The racial and ethnic diversity of the enrolled sample is a meaningful strength relative to most digital mental health trials, where White participants are overrepresented [30]. The use of validated, standardized clinical outcome measures administered as part of routine measurement-based care, rather than research-specific protocols, strengthens the external validity of the clinical findings and reflects real-world practice conditions. The prespecified analysis plan, independent data analysis, and documented conflict of interest safeguards further support the integrity of the reported findings.

Several limitations must be considered when interpreting these results. Most importantly, the retrospective observational design without a control condition precludes any causal inference. The observed within-measure improvements in depression, anxiety, and well-being are consistent with a platform effect but cannot be distinguished from natural symptom recovery, regression to the mean, or the effects of other concurrent support that adolescents and young adults may have been receiving during the observation window. Future studies using a waitlist control, a matched comparison, or quasi-experimental designs are necessary to establish whether platform engagement produces improvements beyond what would be expected in the absence of the platform. Second, only 1437 of 5673 (25.3%) enrolled adolescents and young adults had paired outcome data, and all clinical outcome results reported here should be understood as an exploratory, complete-case analysis of this retained subcohort rather than an estimate of platform-wide effectiveness. Baseline symptom severity did not differ significantly between adolescents and young adults with and without follow-up data across any of the 3 outcome measures (all *P*>.15), indicating that adolescents and young adults with and without follow-up data did not differ in measured symptom severity at intake. However, follow-up survey eligibility was contingent on recent platform engagement, as surveys were administered only to adolescents and young adults who had communicated with their mentor during the preceding 30 days. Consequently, the survey-paired analytic sample may overrepresent adolescents and young adults who remained actively engaged with the platform and underrepresent adolescents and young adults who disengaged early, which limits the generalizability of the clinical outcome findings to the full enrolled population. A sensitivity analysis bounding MCID rates against the full enrolled cohort, rather than the paired analytic sample, produced estimates ranging from 2.9% (168/5673) to 4.2% (237/5673) to 77.6% (4404/5673) to 78.8% (4473/5673) depending on the assumed outcome for participants without paired data, underscoring that the true population-level rate of clinically meaningful improvement cannot be precisely estimated from the available data. Clinical outcome findings should therefore be interpreted as reflecting adolescents and young adults who remained sufficiently engaged to contribute follow-up data rather than the full enrolled population.

Third, interpretation of the clinical outcome analyses is impacted by the platform’s conditional survey routing. Although pre-post analyses were restricted to adolescents and young adults with paired scores on the same instrument, observed changes may reflect both within-person symptom change and shifts in which adolescents and young adults were routed to a given measure. Comparisons across outcome measures should be interpreted with caution, and future studies should administer a consistent battery of outcome measures at each assessment point to enable more rigorous longitudinal modeling. Fourth, the enrolled sample was predominantly female (4600/5673, 81.1%), which limits the generalizability of the findings to male and nonbinary adolescents, groups that are simultaneously underrepresented in mental health service use and at elevated risk of unmet need [32]. Future studies should prioritize outreach strategies and platform design features that improve engagement among male and nonbinary adolescents, a population underrepresented in mental health service use and research samples despite an elevated risk of unmet need. Fifth, the sample spans a wide age range (13‐26 y), encompassing meaningfully different developmental contexts across early, middle, and late adolescence into young adulthood. Age was examined as a continuous predictor of engagement, but because it was not significantly associated with engagement outcomes, the present analyses did not further evaluate differences across developmental age subgroups. Future research should investigate whether outcomes vary across these developmental stages.

### Conclusions

Adolescents and young adults enrolled in a commercially available text-based peer support platform presented with moderate-to-severe mental health symptom burden and demonstrated statistically significant within-measure improvements on paired assessments within the first follow-up window. Approximately one-third of adolescents and young adults who remained actively engaged with the platform achieved clinically meaningful improvement on each measure (ie, PHQ-9, GAD-7, and WHO-5). As an exploratory complete-case finding, this finding is preliminary and should not be extrapolated to the full enrolled cohort. The platform enrolled a racially and ethnically diverse sample, with female adolescents and young adults and those residing in geographically underserved communities engaging most intensively. Considering the lack of a control group, these findings support preliminary evidence of the feasibility of text-based peer support as a scalable approach to expanding mental health access for adolescents and young adults who would otherwise go underserved. Prospective studies with control conditions are needed to establish causal efficacy and to identify the platform features and mentor behaviors most effective at sustaining engagement among adolescents.

## Supplementary material

10.2196/108377 Multimedia Appendix 1 Clinical outcomes questionnaire survey logic, including the initial question on participants’ emotional state and the conditional administration of the Patient Health Questionnaire-9 (PHQ-9), Generalized Anxiety Disorder-7 (GAD-7), or World Health Organization-5 Well-Being Index (WHO-5) questionnaires based on their responses.

10.2196/108377 Checklist 1 STROBE checklist.

## Abbreviations

BRANY: Biomedical Research Alliance of New York
GAD-7: Generalized Anxiety Disorder-7
HPSA: Health Professional Shortage Area
MCID: minimum clinically important difference
PHQ-9: Patient Health Questionnaire-9
STROBE: Strengthening the Reporting of Observational Studies in Epidemiology
WHO-5: World Health Organization-Five Well-Being Index
