# Supplementary material for: Engagement Patterns and Clinical Outcomes Among Adolescents and Young Adults Enrolled in the Somethings Digital Peer Support Platform: Retrospective Observational Study
Source: JMIR Form Res. 2026 Nov 3;10:e108377. doi: 10.2196/108377 (PMC13730504; doi:10.2196/108377)
Supplement: Supplementary file 1 [file formative-v10-e108377-s001.docx]

**Multimedia Appendix 1**

**Supplementary Material 1.**

Below presents the clinical outcomes questionnaire survey logic.

1. Which one’s closest to how you're feeling?

- Feeling down / low energy (Sad, tired, or unmotivated)
- Feeling stressed or anxious (Worried, tense, or on edge)
- Feeling okay overall (Mostly fine, just day-to-day stuff)

*If “feeling down/low energy,” serve PHQ-9A*

2. In the last two weeks, how often have you…
   a. Felt down, depressed, irritable, or hopeless?
   b. Felt little interest or pleasure in doing things?
   c. Had trouble falling asleep, staying asleep, or sleeping too much?
   d. Had poor appetite, weight loss, or overeating?
   e. Felt tired or had little energy?
   f. Felt bad about yourself — or that you are a failure or have let yourself or your family down?
   g. Had trouble concentrating on things like schoolwork, reading, or watching TV?
   h. Been moving or speaking so slowly that others noticed, or been fidgety/restless?
   i. Had thoughts that you would be better off dead or of hurting yourself?

- 0 not at all
- 1 several days
- 2 more than half the days
- 3 nearly every day

*If “feeling stressed/anxious” serve GAD-7*

3. In the last two weeks, how often have you…
   a. Felt nervous, anxious, or on edge?
   b. Been unable to stop or control worrying?
   c. Worried too much about different things?
   d. Had trouble relaxing?
   e. Been so restless that it is hard to sit still?
   f. Become easily annoyed or irritable?
   g. Felt afraid, as if something awful might happen?

*If “feeling okay overall” serve WHO-5*

4. **In the last two weeks, how often have you…**
   a. Felt cheerful and in good spirits?
   b. Felt calm and relaxed?
   c. Felt active and vigorous?
   d. Woken up feeling fresh and rested?
   e. Felt that your daily life has been filled with things that interest you?

**Supplemental Material 2.**

Models A and B were re-estimated with racial/ethnic categories reordered alphabetically, resulting in Asian adolescents and young adults (n = 243, 4.6% of the sample) serving as the reference category in place of White non-Hispanic adolescents and young adults used in the primary specification (Supplemental Table S2). Under this specification, no racial/ethnic group differed significantly from Asian adolescents and young adults in communication days per month.

Supplemental Table S2. OLS regression: predictors of communication days per month.

|  | Model A  (n=5,453) | | Model B  (n=475) | |
| --- | --- | --- | --- | --- |
|  | β (SE) | p | β (SE) | p |
| Age (years, centered) | −0.05 (0.04) | .274 | 0.17 (0.14) | .234 |
| Sex (ref: male) |  |  |  |  |
| Female | 0.70 (0.22) | .002 | -1.15 (0.78) | .140 |
| Race/ethnicity (ref: Asian) |  |  |  |  |
| Black/African American | -0.14 (0.43) | .750 | -0.41 (1.37) | .766 |
| Hispanic/Latino | 0.23 (0.46) | .617 | -2.19 (1.50) | .143 |
| Other/unknown | -0.57 (0.46) | .208 | -3.06 (1.51) | .043 |
| White (non-Hispanic) | -0.50 (0.42) | .237 | -1.59 (0.37) | .228 |
| HPSA shortage score | -0.11 (0.08) | .183 | — | — |
| Baseline severity (*z*-score) | — | — | 0.04 (0.37) | .914 |
| *n* | 5,453 | | 475 | |
| *R*² | .004 | | .029 | |
| Model B restricted to adolescents and young adults with available HPSA shortage score data. | | | | |

**Supplementary Material 3.**

Supplemental Table S3 presents sensitivity analyses comparing OLS, HC3-robust, and log-transformed specifications for Models A and B. In Model A, the effects of female sex, Hispanic/Latino race/ethnicity, and HPSA shortage score were directionally and statistically consistent across all three specifications. Age was not significant under OLS or HC3 but reached significance under log-transformation (p = .010), reflecting the outcome's right-skew rather than a substantive change in effect. In Model B, none of the demographic, severity, or HPSA effects were significant under OLS or log-transformation. The Other/Unknown race/ethnicity coefficient reached significance under HC3 alone (p = .018) but not under OLS (p = .110) or log-transformation (p = .078), indicating this finding is sensitive to model specification and should be interpreted with caution.

| Table S3. Sensitivity analyses comparing OLS, HC3-robust, and log-transformed regression models. | | | |
| --- | --- | --- | --- |
|  | OLS  β (SE), p | HC3  β (SE), p | Log  β (SE), p |
| **Model A (N=5,453)** | | | |
| Age | −0.05 (0.04), .230 | −0.05 (0.04), .169 | −0.02 (0.01), .010 |
| Female | 0.64 (0.22), .004 | 0.64 (0.23), .004 | 0.12 (0.03), <.001 |
| Black/AA | 0.27 (0.21), .207 | 0.27 (0.21), .210 | 0.02 (0.03), .434 |
| Hispanic/Latino | 0.61 (0.26), .019 | 0.61 (0.27), .026 | 0.09 (0.04), .018 |
| Asian | 0.49 (0.42), .240 | 0.49 (0.38), .201 | 0.10 (0.06), .097 |
| Other/Unknown | −0.11 (0.25), .654 | −0.11 (0.25), .656 | −0.04 (0.04), .279 |
| HPSA shortage | 0.08 (0.02), <.001 | 0.08 (0.02), <.001 | 0.01 (0.002), <.001 |
| R² | .008 | – | .013 |
| **Model B (N=1,080)** | | | |
| Age | 0.03 (0.10), .762 | 0.03 (0.09), .734 | -0.01 (0.02), .640 |
| Female | 0.11 (0.50), .825 | 0.11 (0.52), .831 | 0.03 (0.08), .726 |
| Black/AA | 0.82 (0.46), .072 | 0.82 (0.48), .087 | 0.11 (0.07), .133 |
| Hispanic/Latino | -0.27 (0.63), .673 | -0.27 (0.59), .653 | -0.07 (0.10), .493 |
| Asian | 2.02 (0.88), .022 | 2.02 (1.02), .049 | 0.30 (0.14), .028 |
| Other/Unknown | 0.01 (0.61), .992 | 0.01 (0.63), .992 | -0.06 (0.10), .552 |
| HPSA shortage | 0.04 (0.05), .427 | 0.04 (0.05), .500 | 0.003 (0.01), .699 |
| Baseline severity | 0.10 (0.23), .678 | 0.10 (0.25), .700 | -0.01 (0.04), .735 |
| R² | .030 | – | .030 |
| Note. HC3 = heteroskedasticity-consistent robust standard errors, addressing potential violations of homoskedasticity; Log = outcome log-transformed (log1p) to address right-skew; HC3 models do not produce an R² statistic. Reference categories: male (sex), White non-Hispanic (race/ethnicity). | | | |

**Supplementary Material 4.**

Model B in the main text required at least 2 of 3 baseline clinical measures (PHQ-9, GAD-7, WHO-5) to be non-missing for inclusion of the baseline severity composite (n = 1,080), preserving the z-score as a genuine multi-instrument composite. Two sensitivity analyses relaxed or tightened this threshold: (1) at least 1 of 3 measures non-missing (n = 2,906), and (2) all 3 measures non-missing (n = 475). Model specifications were otherwise identical to the primary Model B (communication days per month regressed on age, sex, race/ethnicity, HPSA shortage score, and baseline severity).

|  | **≥1 Measure (n = 2,906)**  β (SE), p | **All 3 Measures**  **(n = 475)**  β (SE), p |
| --- | --- | --- |
| Age (centered) | -0.08 (0.06), .176 | 0.18 (0.15), .218 |
| Sex (Female vs. Male) | 0.92 (0.32), .004 | -1.15 (0.78), .142 |
| Race: Asian vs. White | 0.38 (0.53), .464 | 1.63 (1.32), .220 |
| Race: Black/African American vs. White | 0.57 (0.29), .055 | 1.19 (0.67), .074 |
| Race: Hispanic/Latino vs. White | 0.89 (0.37), .015 | -0.59 (0.89), .509 |
| Race: Other/Unknown vs. White | 0.29 (0.36), .422 | -1.46 (0.91), .110 |
| Baseline severity (z-score) | -0.51 (0.12), <.001 | 0.03 (0.37), .927 |
| HPSA shortage score | 0.15 (0.03), <.001 | 0.02 (0.06), .705 |
| Adjusted R² | 0.022 | 0.013 |

**Supplementary Material 5.**

As a sensitivity check on the potential impact of missing outcome data, we recalculated clinically meaningful improvement (MCID) rates under two bounding assumptions, using the full enrolled cohort (n = 5,673) as the denominator in place of the paired analytic sample (n = 1,437, 25.3%). Under a conservative (worst-case) bound, in which enrolled participants without paired outcome data were assumed not to have met MCID, estimated rates fell to 4.2% (PHQ-9), 3.9% (GAD-7), and 3.0% (WHO-5), compared to complete-case rates of 31.9%, 36.6%, and 38.0%, respectively. Under a liberal (best-case) bound, in which those same participants were assumed to have met MCID, estimated rates rose to 78.8% (PHQ-9), 78.6% (GAD-7), and 77.6% (WHO-5). Both bounds used a uniform paired/not-paired split (1,437/4,236) applied consistently across all three measures, rather than a per-instrument split, to avoid conflating true attrition with the platform's conditional survey routing across instruments.

| **Measure** | **Complete-Case** | **Worst-Case** | **Best-Case** |
| --- | --- | --- | --- |
| **PHQ-9** | 31.9% | 4.2% | 78.8% |
| **GAD-7** | 36.6% | 3.9% | 78.6% |
| **WHO-5** | 38.0% | 3.0% | 77.6% |
